# Anatomically constrained liver CT anomaly detection using healthy priors with diffusion-based inpainting

**DOI:** 10.21203/rs.3.rs-8563123/v1

**Published:** 2026-01-16

**Authors:** Eshan Joshi, Yongyi Shi, Albert Montillo, Matthew Lewis, Ron Peshock, Ge Wang, Samuel Achilefu

**Affiliations:** University of Texas Southwestern Medical Center; Rensselaer Polytechnic Institute; University of Texas Southwestern Medical Center; University of Texas Southwestern Medical Center; University of Texas Southwestern Medical Center; Rensselaer Polytechnic Institute; University of Texas Southwestern Medical Center

**Keywords:** computed tomography, anomaly detection, diffusion models, unsupervised learning, generative artifcial intelligence

## Abstract

Detecting subtle focal liver lesions on abdominal computed tomography (CT) is challenging in routine clinical practice, especially for small, low-contrast, or morphologically heterogeneous tumors acquired under variable protocols. While fully supervised liver tumor segmentation can achieve high accuracy, it requires pixel-level annotations that limit scalability and generalizability. Reconstruction-based anomaly detectors trained without hepatic anatomical constraints reduce label burden but are sensitive to textural variability, contrast-phase differences, and produce noisy, unstable boundaries. We introduce an anatomically constrained, four-stage pipeline for liver CT anomaly detection: (1) a denoising diffusion probabilistic model (DDPM) trained on unremarkable axial slices to learn a healthy prior; (2) diffusion-based inpainting within an automatically segmented whole-liver mask to generate pseudo-normal liver appearance; (3) a compact encoder–decoder trained with a liver-masked, mean squared error loss to reconstruct healthy liver tissue from paired original and inpainted inputs; and (4) a liver-scoped difference map between the original and reconstructed healthy CT slices as the final anomaly score for localization. Trained exclusively on > 13,000 healthy CT slices and evaluated on 1,000 abnormal CT slices from 109 Liver Tumor Segmentation (LiTS) benchmark patients, the method achieves Dice 0.596, intersection-over-union 0.482, area under the receiver operating characteristic curve 0.861, and 95th percentile Hausdorff distance 80.5 pixels (px). Performance improves with lesion size, with a Dice score of 0.796 for the largest quartile. Anchoring anomaly detection to hepatic anatomy with a stable healthy prior yields data-efficient liver lesion localization suitable for CT triage and prioritization.

## Introduction

Accurate detection of focal liver lesions on abdominal CT is essential for timely diagnosis and treatment planning, yet manual identification remains difficult in routine clinical practice. The increasing sensitivity of modern cross-sectional imaging has revealed a high prevalence of incidental hepatic findings, with lesions detected in approximately one-third of middle-aged and older adults [1]. While most are benign, distinguishing clinically significant lesions from incidentalomas is challenging and can lead to unnecessary follow-up imaging, invasive procedures, and patient anxiety [1]. Small lesions, low lesion-to-liver contrast, heterogeneous lesion morphology, and variability in acquisition protocols can make abnormalities visually subtle, further increasing the risk of missed or delayed diagnoses in high-volume clinical workflows [2–6]. Automated tools that can reliably identify and prioritize conspicuous lesions for review could help radiologists focus attention where it is most needed and reduce missed significant pathology and the burden of unnecessary workup of benign incidentalomas.

While these challenges motivate computational assistance, prior automation approaches exhibit important limitations. Fully supervised segmentation can achieve liver tumor delineation on CT, but it depends on large, pixel-level annotations that are expensive to curate, and are often inconsistent across institutions, scanners, and contrast phases [7]. Variability in annotation protocols and interobserver inconsistency further complicate model training, limit generalizability, and reduce the scalability and portability of fully supervised models [7–11]. Unsupervised or weakly supervised reconstruction detectors reduce annotation burden but, without liver-specific scoping, often learn nonhepatic confounds, suffer phase-related domain shift, and produce unstable thresholds and noisy boundaries, especially for small, low-contrast lesions [12–14]. Classification-only approaches (at the image or patch level) localize poorly and can overcall artifacts at organ edges [8, 10]. Together, these factors have limited generalization and reliability in real-world clinical workflows, motivating an anatomically scoped pipeline with a stable healthy prior and minimal, targeted supervision [7, 8].

Automated anomaly detection offers a complementary approach that models healthy tissue as a normative distribution and identifies deviations as suspicious outliers [12–19]. DDPMs learn this normative distribution by modeling the data-generating process through iterative denoising, enabling them to sample from the learned healthy tissue manifold [10–11, 14, 19, 20–22]. When applied to inpainting, these models can generate pseudo-normal references by regenerating masked regions under the healthy prior [19]. However, existing approaches typically require prior knowledge of anomaly location to define the inpainting mask, creating a circular dependency where detection depends on already knowing what to detect [10, 16, 19, 21]. More generally, naive reconstruction-based approaches struggle in the liver because of the considerable textural variability of hepatic parenchyma, phase-related intensity differences, and boundary artifacts [12]. Without anatomical constraints, such models may overfit to background structures or imaging noise. In addition, inadequate spatial scoping, or failing to constrain each stage to the appropriate anatomy, can introduce train–test domain shifts that further degrade performance [8].

In this work, we introduce a liver-scoped anomaly detection pipeline that aims to combine the strengths of diffusion-based healthy priors and reconstruction-based anomaly mapping while explicitly anchoring each processing stage to hepatic anatomy. A DDPM is trained on full axial CT slices from patients without abdominal pathology to learn a healthy abdominal prior. This prior is then used for diffusion-based inpainting restricted to automatically segmented whole-liver masks, generating pseudo-normal liver appearance while preserving extrahepatic context. Next, a compact convolutional encoder–decoder is trained, using only healthy data and a masked loss over the liver region, to reconstruct healthy liver tissue from paired original and inpainted inputs. During inference, the absolute difference between the original and reconstructed images within the liver mask forms an anatomically scoped anomaly map, which undergoes light post-processing. We evaluate this pipeline on the LiTS benchmark, reporting Dice similarity coefficient (Dice), intersection over union (IoU), 95th percentile Hausdorff distance (HD95), area under the receiver operating characteristic (AUROC) curve, and tumor-size-stratified performance to reflect practical triage utility [23]. We also compare it against a healthy-image-trained U-Net autoencoder baseline and an inpainting-only anomaly score to quantify the contributions of the healthy reconstruction and post-processing steps.

Overall, our results show that anchoring anomaly detection to hepatic anatomy through diffusion-based pseudo-normal generation, liver-masked healthy reconstruction, and anatomically scoped anomaly maps yields strong pixel-level discrimination, lesion localization, and reduced false positives using only healthy training data. These findings demonstrate that our method functions as intended, with accuracy increasing for larger lesions, generalization remaining stable across patients, and healthy reconstruction proving essential for achieving precise and well-localized anomaly maps. The computational profile supports near real-time inference when deployed on standard institutional GPU hardware, aligning the method with high-throughput CT triage workflows. Inference speed is reported for a fixed hardware configuration and reflects the anomaly-scoring pipeline, with detailed implementation and hardware specifications provided in the Methods section. The approach is intended as a clinical decision-support or triage tool that flags abnormal liver regions for focused manual review rather than replacing radiologist interpretation.

## Results

### Summary of approach

**Figure 1:**
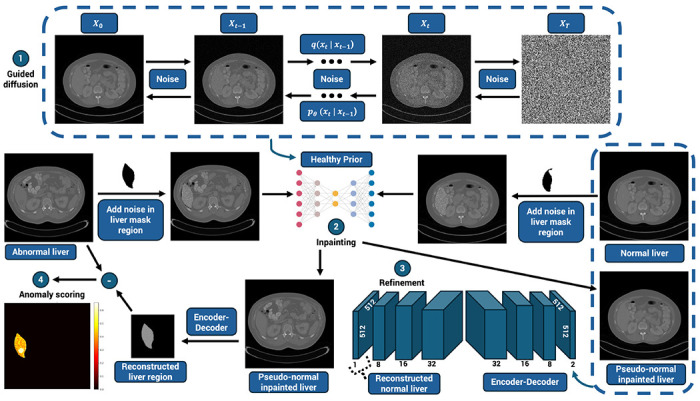
Overview of the four-stage, anatomically scoped anomaly-detection pipeline. (1) A diffusion model generates pseudo-normal liver candidates from full CT slices while preserving global anatomical context. (2) Diffusion-based inpainting is applied within the whole-liver mask to create a pseudo-normal liver region guided by the anatomical context of the original image. (3) A compact encoder–decoder reconstructs healthy liver appearance from paired original and inpainted slices using a liver-masked loss. (4) A liver-scoped anomaly map is computed as the absolute pixel-wise difference between the original and reconstructed images, followed by light post-processing to produce the final tumor localization.

Our anatomically scoped pipeline, designed for liver anomaly detection, addresses key limitations of prior reconstruction-based approaches by anchoring every modeling step to hepatic anatomy and requiring only healthy training data. A 2D slice-based approach was adopted to reduce computational cost, improve data efficiency under heterogeneous acquisition conditions, and enable near–real-time inference compatible with clinical triage workflows. Whole-liver masks were automatically generated for each CT slice using a pretrained segmentation model, TotalSegmentator, ensuring consistent spatial scoping across subjects [24]. The pipeline comprises four stages that progressively narrow from the full CT slice to the liver parenchyma (Fig. 1).

First, a DDPM is trained on full axial CT slices to learn the distribution of healthy abdominal appearance while preserving global organ layout, patient habitus, and acquisition characteristics. At inference, iterative denoising from Gaussian noise produces pseudo-normal candidates that predict a healthy slice, preserving global anatomy without applying a liver mask. Second, diffusion-based inpainting is applied only within the automatically segmented liver, regenerating healthy liver appearance while preserving all extrahepatic structures. Third, a compact encoder–decoder is trained to reconstruct healthy liver tissue from paired original and inpainted slices using a liver-masked loss, producing a stable anatomy-specific healthy prior while avoiding confounds from background structures. Fourth, a liver-scoped anomaly map is computed as the pixel-wise absolute difference between the original and reconstructed slices, followed by light post-processing to suppress speckle, remove small artifacts, and stabilize boundaries across lesion sizes. This structured progression from whole-slice diffusion modeling to liver-only anomaly scoring enables data-efficient learning of healthy liver appearance and the detection of deviations with minimal supervision. The method is designed as a decision-support and triage tool rather than a volumetric segmentation system, prioritizing rapid identification of suspicious liver regions over precise 3D tumor delineation. The following sections quantify the performance of this pipeline across lesion sizes, patient subsets, and baseline comparisons.

### Overall anomaly-detection performance

**Figure 2:**
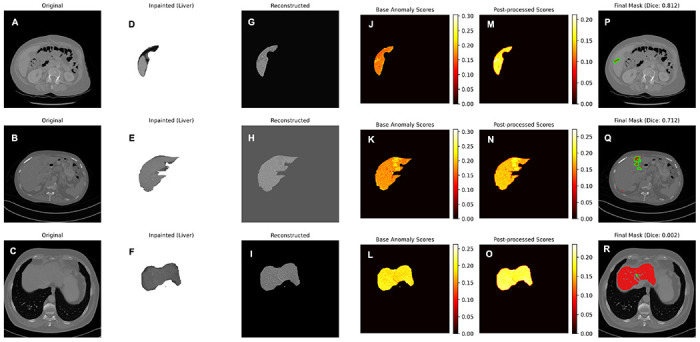
Intermediate anomaly maps across lesion difficulty tiers. Representative slices sampled from high- (top row), mid- (middle row), and low-performing (bottom row) cases (based on Dice ranks) are shown with the original CT (panels A-C), liver-inpainted input (panels D-F), encoder-decoder reconstruction (panels G-I), base (panels J-L)and post-processed anomaly maps (panels M-O), and the final prediction overlaid on the CT with the ground-truth tumor contour (panels P-R). All anomaly maps are liver-masked to emphasize organ-restricted predictions. In the final column, model predictions are shown in red, and contours, shown in green, highlight true tumor boundaries.

**Figure 3:**
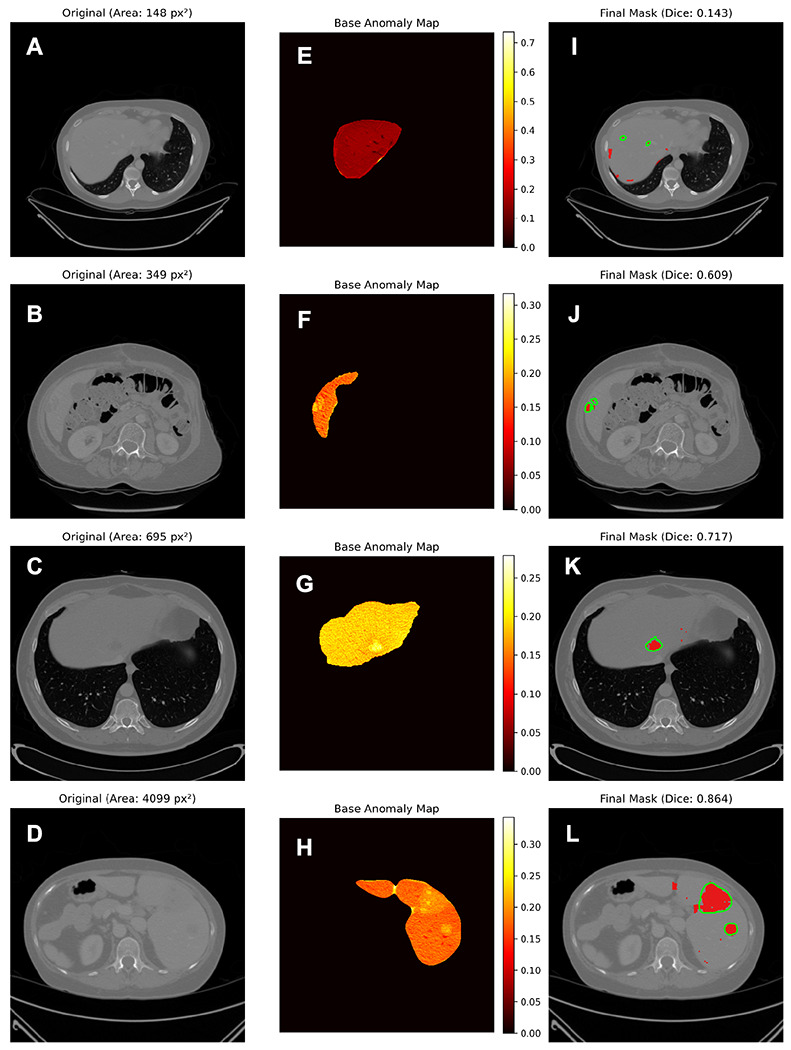
Qualitative detection examples across tumor sizes. Rows illustrate large focal lesions (bottom row), small focal lesions (third row), and difficult cases (top two rows), each showing the original CT (panels A-D), anomaly heatmap (panels E-H), and final segmentation overlay (panels I-L). In the final column, model predictions are shown in red, and contours, shown in green, highlight true tumor boundaries.

We evaluated the anatomically scoped pipeline on 1,000 abnormal slices from 109 LiTS benchmark patients using liver-masked Dice, IoU, HD95, and voxel-wise AUROC. The method achieved a mean Dice score of 0.596 (95% CI: 0.578–0.614), IoU of 0.482 (95% CI: 0.465–0.500), and HD95 of 80.5 px (95% CI: 76.6–84.5). Pixel-level discrimination within the liver mask reached an AUROC of 0.861 (95% CI: 0.852–0.870). These results indicate that the combination of diffusion-guided pseudo-normal reconstruction, liver-scoped reconstruction, and light post-processing produces spatially coherent anomaly maps with moderate boundary error and strong pixel-level differentiation between tumor and healthy parenchyma. Representative examples across the performance spectrum are shown in Fig. 2 for high-, mid-, and low-performing slices (based on Dice ranks), illustrating how diffusion-based inpainting and healthy reconstruction jointly shape the anomaly signal. Examples spanning large, small, and diffuse lesions (Fig. 3) highlight the range of tumor appearances handled by our method. Large and well-defined lesions yield focused anomaly responses with clear boundaries, whereas small, low-contrast, or diffuse lesions produce more subtle or fragmented maps.

### Tumor size effects and ROC stratification

**Figure 4:**
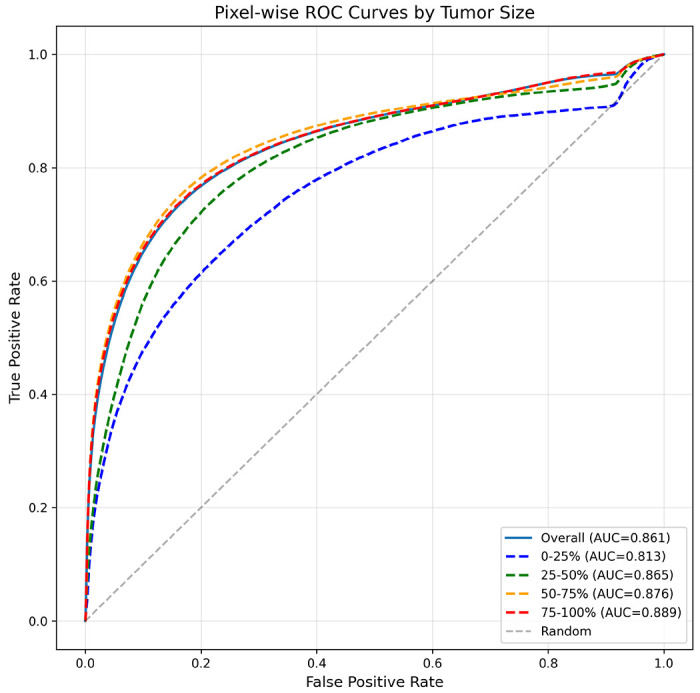
Pixel-wise ROC curves stratified by tumor size. ROC curves compare per-pixel anomaly classification performance across quartiles of tumor area (smallest to largest). Points indicate the F1-optimized thresholds on validation data, highlighting the size-dependent trade-off between sensitivity and specificity.

**Figure 5:**
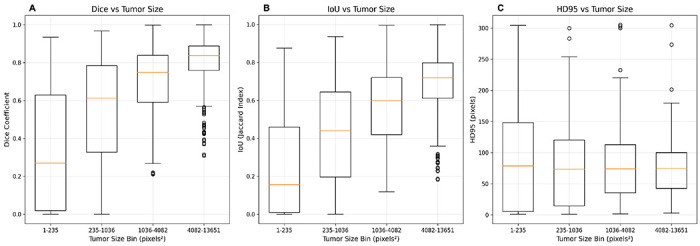
Lesion-size-stratified metrics. Box plots summarize Dice (panel A), IoU (panel B), and HD95 (panel C) for quartiles of tumor size (in pixels^2^), with paired IoU and HD95 plots illustrating how overlap and boundary accuracy improve as lesions grow larger. Values are liver-masked to prevent extrahepatic bias; whiskers reflect median with IQR.

Performance improved systematically with lesion size (Fig. 4, Table 1). Lesion size is reported in pixel area rather than physical units to reflect the spatial support available to the slice-based model and to avoid confounding from variable in-plane resolution across scans. Dice increased from 0.339 in the smallest quartile (1–235 px^2^) to 0.796 in the largest (4,082–13,651 px^2^), and IoU rose from 0.253 to 0.682 across the same bins. Pixel-wise AUROC increased from 0.813 to 0.889 across quartiles. This trend reflects the expected dependence of reconstruction-based anomaly detectors on spatial footprint, with larger lesions producing stronger, more stable difference maps and cleaner boundaries than the weaker and more variable responses from very small foci. ROC curves mirrored these patterns (Fig. 5), with AUC values increasing from the smallest (0.813) to the largest quartile (0.889). These ROC trends align with the overlap metrics, showing more reliable voxel-level separation for larger or more conspicuous lesions. Qualitative examples in Fig. 4 illustrate this behavior, showing that larger lesions exhibit strong, localized responses and clear boundaries, whereas very small lesions show greater variability in signal intensity and shape, consistent with size-stratified performance.

### Cross-validation and runtime characteristics

**Figure 6:**
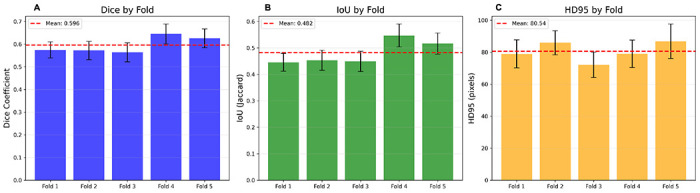
Cross-validation fold performance with 95% CI. Bars show fold-wise mean Dice (panel A), IoU (panel B), and HD95 (panel C) for the patient-grouped, 5-fold cross-validation. Error bars denote 95% confidence intervals of the fold mean (t-distribution), and dashed horizontal lines mark the overall mean across folds.

Patient-grouped K-fold cross-validation (K = 5) on the abnormal dataset demonstrated consistent performance across patient subsets with heterogeneous tumor burdens. Fold-wise Dice ranged 0.572–0.645 (mean 0.596, SD 0.294), IoU ranged 0.445–0.547 (mean 0.482, SD 0.280), and HD95 ranged 70.1–86.8 px (mean 80.5 px, SD 62.7) (Fig. 6). Fold-to-fold variation was modest relative to lesion heterogeneity in the dataset, indicating stable performance across patient groups with differing tumor burdens and appearances.

Median inference time for reconstruction, post-processing, and anomaly scoring was 0.52 seconds per slice (IQR 0.35–0.68), corresponding to approximately 31 slices per minute. Most slices processed in under 1 second, supporting triage throughput. Diffusion-based inpainting, which was performed prior to inference, required approximately 26 seconds per slice. The resulting inference-time component is compatible with interactive triage use cases or background pre-processing in clinical Picture Archiving and Communication System (PACS) workflows. These results align with the design goal of separating computationally intensive pseudo-normal generation from fast per-slice inference.

### Baseline comparisons and ablation analyses

**Figure 7:**
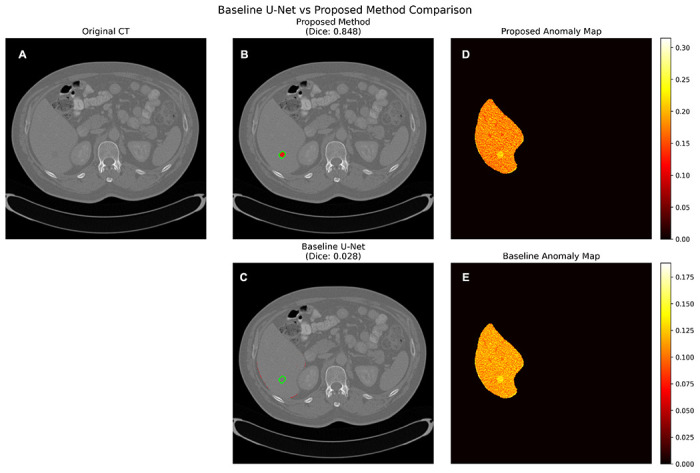
Baseline U-Net comparison. Side-by-side qualitative comparison between the proposed encoder–decoder difference model and a U-Net autoencoder baseline on the same slice. Each row shows the original CT (panel A), thresholded prediction overlay (using proposed method in panel B and baseline U-Net in panel C), and anomaly map within the liver mask (using proposed method in panel D and baseline U-Net in panel E). Model predictions are shown in red, and contours, shown in green, highlight true tumor boundaries. The U-Net baseline struggles with diffuse false positives, resulting in a lower Dice.

Our method outperformed two key reconstruction-based baselines. Compared to a healthy-trained U-Net autoencoder, it achieved a > 700% improvement in Dice (example shown in Fig. 7), demonstrating the value of anatomically scoped reconstruction over generic slice-level reconstruction. Relative to inpainting-only detection (|*x_original_ − x_inpainted_*|) Dice improved by 166%, showing that learned healthy reconstruction is essential for sharpening boundaries and suppressing false positives compared to simpler alternatives.

To delineate the contributions of individual pipeline components and assess whether performance gains arise from diffusion-based inpainting alone or from subsequent reconstruction and post-processing, we performed targeted ablation studies. Our results confirmed the contribution of healthy reconstruction and post-processing components to overall detection performance (Table 2). Replacing the encoder-decoder healthy reconstruction with the inpainted liver alone reduced Dice by 0.372, indicating that inpainting alone is insufficient for accurate localization. Removing light post-processing reduced Dice from 0.596 to 0.403 and increased HD95 from 80.5 px to 127 px, demonstrating the importance of spatial smoothing and morphological cleanup for boundary stability. Together, these analyses show that each stage of the anatomically scoped pipeline contributes meaningfully to final detection quality, with post-processing providing small but consistent gains in overlap and boundary stabilization.

## Discussion

This study demonstrates that a diffusion-informed healthy prior with liver-scoped reconstruction and light post-processing provides clinically meaningful anomaly detection for liver CT with modest supervision and practical runtime. On the LiTS benchmark, our pipeline achieved moderate-to-high overlap (Dice 0.596, IoU 0.482) and strong pixel-wise discrimination (AUC 0.861) within the liver mask, with acceptable boundary error (HD95 80.5 pixels). These results are comparable to reported inter-reader overlap for liver lesion segmentation and align with the intended role of the method as a triage and decision-support tool, where suspicious liver regions are surfaced for rapid triage without requiring dense annotations [4].

Performance stratified by tumor size reveals a clear size dependency that aligns with clinical experience [4, 25]. Small lesions had lower Dice and IoU, while medium-to-large lesions exhibited substantially better overlap and more stable boundaries. Small lesions remain challenging due to subtle contrast, partial volume effects, and limited spatial support. These trends suggest that diffusion-generated pseudo-normal references stabilize reconstruction errors for medium- to large-sized lesions, while additional strategies are needed to boost sensitivity to subtle foci. This behavior mirrors observations from medical anomaly-detection benchmarks, where liver CT has been found to favor methods that capture low-level feature differences but still struggle with small or subtle anomalies [1, 4, 25]. In practice, such size dependence may be acceptable for a triage system aimed at prioritizing conspicuous lesions and prompting secondary review, provided that future work addresses sensitivity to small, early-stage tumors.

Relative to previous unsupervised and weakly supervised liver lesion detection approaches, the main contribution of this work lies in its anatomically scoped integration of diffusion-based inpainting and healthy reconstruction. Autoencoder-like and adversarial methods for unsupervised tumor segmentation reconstruct lesion-free images and use reconstruction error as an anomaly map, but are typically trained on either full slices or cropped regions without enforcing organ-masked losses or evaluation [12–19]. These methods can learn confounding background patterns, are sensitive to acquisition differences, and often require careful tuning of thresholds or post-processing to suppress false positives near organ boundaries.

Shi et al. recently introduced a diffusion-based anomaly detector for liver CT that uses adaptive thresholding to identify suspicious regions, applies diffusion inpainting within those regions, and computes the anomaly score from the discrepancy between the original and inpainted images, achieving higher AUCs than earlier reconstruction-based methods on two datasets [19]. Our approach is complementary but differs in several respects. First, we anchor several processing stages to hepatic anatomy: the encoder-decoder reconstruction loss, anomaly computation, and evaluation are all constrained by automatically generated whole-liver masks. Second, rather than relying on inpainting differences alone or heuristics such as intensity or density cues to define anomalous regions, we use the automatically segmented whole-liver mask itself to guide inpainting and train a compact encoder–decoder on healthy data to reconstruct liver parenchyma from paired original and inpainted inputs, yielding sharper boundaries and more stable anomaly scores. Third, we explicitly quantify the contribution of healthy reconstruction and post-processing through ablation studies, providing tumor-size-stratified metrics and cross-validated performance on LiTS, which facilitates comparison across studies and lesion scales.

Diffusion-based anomaly detection has also been explored in other anatomies, such as pancreatic and brain tumor segmentation, where DDPMs are used to generate pseudo-normal references for outlier detection [12–18]. Our results extend this line of work by demonstrating that diffusion priors can be profitably combined with anatomically scoped reconstruction networks for liver CT, leveraging the strengths of both generative modeling and supervised reconstruction while requiring only healthy data for training.

From a systems perspective, the proposed pipeline is compatible with institutional compute resources. Diffusion model training, while computationally intensive, is performed offline and can be amortized over large datasets or periodically updated as acquisition protocols evolve. Inference with the encoder–decoder and post-processing operates at sub-second per-slice latency on a single GPU, making it feasible to generate anomaly heatmaps in the background as CT studies are acquired. In a clinical workflow, these heatmaps and binary masks could be overlaid on standard CT viewers to highlight suspicious liver regions or used to prioritize cases in high-volume reading lists.

We note that our study has some limitations. First, performance on very small lesions remains modest, which is a common challenge for reconstruction-based anomaly detectors that rely on spatially extended intensity differences. Improving sensitivity to small tumors will likely require multi-scale architectures, higher-resolution processing, or targeted training strategies that emphasize subtle lesions. Second, the pipeline depends on the quality of automatically generated liver masks. We use TotalSegmentator for its robustness across scanners and protocols, but segmentation errors could propagate to the anomaly maps. Future work could explore joint refinement of liver masks or soft-mask training. Third, the current implementation is 2D, applied slice-by-slice to 3D volumes. While 2D processing improves computational efficiency and simplifies training, it does not fully exploit inter-slice continuity or 3D lesion morphology. Extending the approach to volumetric diffusion models and 3D reconstruction networks is a natural next step, provided inference times remain compatible with clinical use. Finally, this retrospective analysis evaluates technical performance on LiTS but does not include prospective reader studies. To establish clinical utility, future work should measure the impact of liver-scoped anomaly maps on radiologist accuracy, reading time, and confidence, and examine how to best integrate anomaly scores into existing reporting workflows.

Despite these limitations, the present results suggest that anatomically constrained, diffusion-informed healthy reconstruction offers a promising, data-efficient strategy for liver CT anomaly detection that is well aligned with real-world triage and prioritization needs.

## Materials and Methods

### Study data and annotations

We performed a retrospective analysis of axial abdominal CT scans acquired at the University of Texas Southwestern Medical Center as part of routine clinical care. All imaging data were fully deidentified prior to processing in accordance with institutional guidelines. A total of 911 subjects (525 adult females and 386 adult males) with normal abdominal CT examinations were identified, from which over 13,000 healthy axial slices were extracted for model training. Whole-liver binary masks were generated automatically using TotalSegmentator, a validated deep learning tool for multi-organ CT segmentation that has demonstrated robust performance across diverse scanner vendors and acquisition protocols [24]. We chose TotalSegmentator for its automated, consistent liver segmentation, which enables scalable processing of large datasets without manual annotation while maintaining anatomical accuracy.

### Data for model training

To standardize sampling and reduce redundancy, we processed every third slice and retained only slices with at least 100 liver pixels, yielding 13,141 processed healthy CT slices from 911 unique subjects. These healthy slices were used to: (1) train the diffusion model, and (2) generate 1,000 inpainted pseudo-normal liver slices for training the encoder-decoder. All model training was performed exclusively on healthy data; no abnormal slices were used during training.

### Data for model evaluation

Abnormal slices and tumor masks were obtained from the LiTS benchmark [7]. The dataset comprises 131 contrast-enhanced abdominal CT volumes acquired across seven clinical institutions, with expert liver and tumor annotations provided by trained radiologists. We used 1,000 abnormal slices from 109 unique patients for independent testing, selected to represent varied lesion sizes from small to large. All 1,000 slices were used in patient-grouped 5-fold cross-validation. For inpainting evaluation, we generated 1,000 inpainted, pseudo-normal slices corresponding to these abnormal test images.

Cross-validation was conducted exclusively on the abnormal cohort; all folds contained only abnormal slices. We used patient-grouped K-fold cross-validation (K = 5) on the abnormal cohort, with all slices from a patient kept in the same fold to prevent cross-patient leakage. Since all slices in the abnormal cohort contain tumors (as verified by ground truth masks), stratification by class proportions was not applicable; instead, folds were balanced by ensuring approximately equal numbers of patients and slices across folds.

### Data pre-processing

All volumes were obtained in Neuroimaging Informatics Technology Initiative (NIfTI) format, in which slices were at a native in-plane resolution of 512 × 512 pixels. Each CT slice was intensity-clipped to the range – 1,000 to 1,600 Hounsfield units (HU), covering the full spectrum from air to high-density contrast-enhanced soft tissue. The CT intensities were normalized using a standard HU-to-attenuation mapping based on the water attenuation constant (μ_w_ = 0.192 cm^−1^), producing values in the [−1, 1] range while preserving relative liver soft-tissue contrast [14, 19, 26]. Liver and tumor masks remained strictly aligned to the resampled images at the fixed in-plane resolution.

The liver mask was used consistently throughout the pipeline to define the anatomical scope of all learning, inference, and evaluation steps. Specifically, it delineated (1) the loss region for training the encoder–decoder, ensuring that healthy reconstruction was optimized only within hepatic parenchyma; (2) the region over which anomaly scores were computed at inference, preventing contributions from extrahepatic structures; and (3) the evaluation region, such that all reported performance metrics reflect detection accuracy within the liver. This consistent liver-level scoping enforces anatomical relevance and reduces background confounds across all stages of the pipeline.

### Model architectures

Our proposed method (see Fig. 1) is comprised of three models:

#### (1) Diffusion model

A DDPM was implemented using the guided-diffusion library and trained on full 2D CT slices to model the distribution of healthy abdominal anatomy [26, 27]. The model uses a U-Net backbone with multi-scale feature layers, cosine noise scheduling, and classifier-free guidance to stabilize sampling. During inference, image generation proceeds by iteratively denoising from Gaussian noise through T = 250 reverse-diffusion steps, producing pseudo-normal CT slices. Because no liver mask is applied at this stage, the model retains whole-slice contextual consistency that is important for downstream appearance modeling.

#### (2) RePaint inpainting model

To isolate healthy liver appearance from surrounding anatomy, we apply RePaint, a diffusion-based inpainting method that resamples only within the segmented liver region [21]. The full CT slice and the liver mask are provided to the model, which repeatedly alternates between (a) constrained forward noising of the liver region and (b) guided denoising using the diffusion prior. Our settings include T = 250 diffusion steps with r = 10 resampling cycles. This process produces a pseudo-normal liver reconstruction that is spatially aligned with the original CT slice while preserving unchanged background anatomy. The resulting inpainted liver appearance serves as the healthy reference target for supervised reconstruction.

#### (3) Encoder-Decoder for healthy reconstruction

A lightweight convolutional encoder–decoder is trained to reconstruct healthy liver tissue from paired inputs consisting of the original CT slice and the pseudo-normal CT slice containing the inpainted liver region. The encoder uses sequential Conv–BatchNorm–ReLU blocks with progressively increasing channel depth (8→16→32), while the decoder mirrors this structure (32→16→8→1) with transposed convolutions for upsampling. Dropout layers provide regularization and help stabilize training. The network is optimized using a masked MSE loss applied only within the liver mask, forcing the model to learn a stable, anatomically coherent healthy prior without influence from background structures. During inference, the absolute pixel-wise difference |*x_original_ − x_reconstrucated_*| within the liver mask forms the anomaly map used for lesion localization. The network architecture was chosen heuristically to balance capacity, stability, and speed after exploratory trials.

### Inference and post-processing

**Figure 8:**
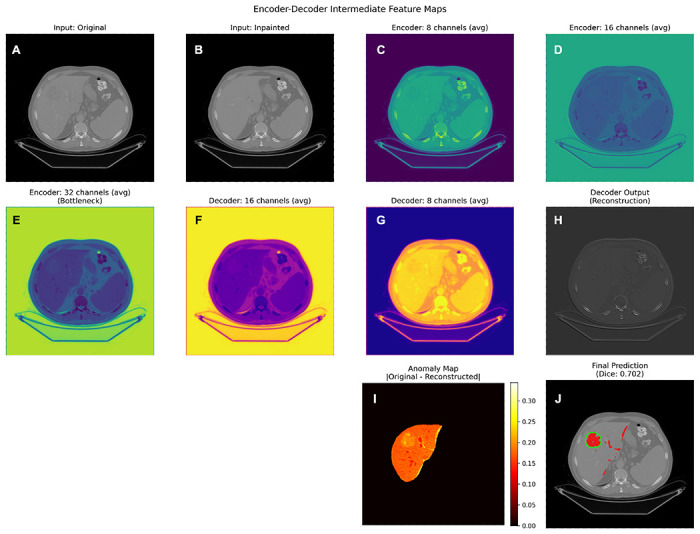
Encoder–decoder intermediate feature maps. For a mid-performing slice, panels visualize the original/inpainted inputs (panels A and B), averaged encoder features (8–32 channels) in panels C through E, decoder activations (16–8 channels) in panels F and G, reconstruction output (panel H), liver-masked anomaly map (panel I), and final prediction overlay (panel J). This highlights how localized deviations emerge as features propagate through the bottleneck. In the final image, model predictions are shown in red, and contours, shown in green, highlight true tumor boundaries.

At inference, the encoder–decoder reconstructs the liver from concatenated inputs (see Fig. 8 for intermediate maps produced by the encoder-decoder). Base anomaly maps are |*x_original_ — x_reconstructed_*| within the liver and define the anomaly signal. The liver-masked difference map is smoothed, edge-suppressed, morphologically cleaned, and thresholded. To stabilize predictions across scans and lesion sizes, we apply light spatial smoothing, mild suppression near the liver boundary, and small morphological opening/closing to reduce speckle and fill pinholes. The optimal threshold for anomaly scoring is chosen as the threshold value that achieves the maximal F1-score during the calculation of the AUC.

### Evaluation metrics and analyses

We report Dice similarity coefficient, IoU, HD95, and AUROC, all computed within the liver mask. To reflect clinical utility across lesion scales, we stratify performance by per-image tumor area percentiles (0–25–50–75–100%). Cross-validation uses patient-grouped K-fold (K = 5), grouped by patient to prevent cross-patient leakage.

We also compared against a standard U-Net autoencoder with skip connections that reconstructs the original slice from concatenated original and inpainted inputs (2→1 channels). The baseline uses a three-level encoder-decoder (32 base channels, scaling to 256 in the bottleneck) and was trained with masked MSE loss on healthy liver tissue. At inference, anomaly scores are computed pixel-wise as |*x_original_ — x_reconstructed_*|within the liver mask and thresholded using F1-scoring to produce binary predictions, providing a direct reconstruction-based comparison without the multi-stage anatomical scoping of the proposed pipeline.

### Ablation studies

Ablation studies were conducted by selectively removing or modifying individual components of the pipeline while keeping all other training, inference, and evaluation settings fixed. Specifically, we evaluated variants that omitted the encoder–decoder healthy reconstruction stage and variants that disabled light post-processing, allowing the contribution of each component to be assessed under identical data splits, thresholds, and hardware configurations.

### Implementation details

All experiments were conducted in Python 3.8 using PyTorch (version 2.2.2). Whole-liver masks were generated with TotalSegmentator [24]. Pseudo-normal references were produced using the guided-diffusion library [6] and liver-region inpainting used RePaint [21]. Abnormal images and tumor masks were obtained from LiTS [8]. Random seeds were fixed for data splits, and both losses and metrics were logged each epoch.

Diffusion model training required approximately 2.5 days to complete 1 million iterations on two compute nodes equipped with 64 physical Intel CPU cores and one NVIDIA H100 GPU (80 GB) each using a batch size of 4, a learning rate of 1×10^™4^, and an exponential-moving-average (EMA) model for stability, with a momentum of 0.999. Inpainting runs (healthy and abnormal cohorts) were executed on a single compute node with 64 physical Intel CPU cores and one NVIDIA H100 GPU (80 GB) using a custom-built, multi-GPU script. The encoder–decoder trained in roughly 30 minutes on a node with 64 physical Intel CPU cores and one NVIDIA H100 GPU (80 GB). At inference, the pipeline processes a CT slice in less than a second on a single node with 36 physical Intel CPU cores and one NVIDIA Tesla P40 GPU (24 GB), including reconstruction and post-processing.

Visualizations were produced in Python 3.8 using the Matplotlib library for preprocessing and overlay generation, and figures were assembled and formatted in Microsoft PowerPoint.

## Conclusion

This study presents an anatomically-informed, diffusion-based strategy for anomaly detection in liver CT that requires only healthy training data and automatically derived liver masks. By combining whole-slice diffusion modeling, liver-restricted inpainting, a compact healthy-reconstruction network, and liver-scoped anomaly mapping, the approach delivers consistent lesion localization across heterogeneous tumor appearances, while maintaining practical inference speed. On the LiTS benchmark, the method achieves clinically meaningful performance (Dice 0.596, IoU 0.482, AUC 0.861) with substantial gains over standard reconstruction-based baselines and clear improvements for medium-to-large lesions. The results demonstrate that anchoring anomaly detection to hepatic anatomy and leveraging pseudo-normal image generation can reduce false positives, stabilize boundaries, and enable data-efficient lesion localization suitable for CT triage and prioritization. Challenges remain for very small lesions and fully capturing 3D lesion morphology; however, the framework provides a scalable foundation for such future extensions in volumetric modeling, small-lesion sensitivity, and domain adaptation. Overall, the findings highlight the value of anatomically constrained reconstruction for advancing practical, generalizable anomaly detection in abdominal CT.

## Figures and Tables

**Table 1:** Performance stratified by tumor size quartile on the LiTS abnormal cohort. Metrics include Dice, IoU, HD95, and pixel-wise AUC, with lesion counts and area ranges shown for each bin. Larger lesions yield higher overlap scores and slightly improved voxel-level discrimination.

Tumor Percentile	Mean Dice (SD, 95% CI)	Mean IoU (SD, 95% CI)	Mean HD95 (SD, 95% CI)	Mean AUROC (SD, 95% CI)
0–25% (n = 250, 1–235 px)	0.339 (0.312, 0.300–0.378)	0.253 (0.262, 0.220–0.286)	88.3 px (78.1, 78.2–98.4)	0.813 (0.215, 0.786–0.840)
25–50% (n = 250, 235–1,036 px)	0.557 (0.271, 0.523–0.591)	0.434 (0.261, 0.402–0.467)	77.5 px (64.2, 69.5–85.6)	0.865 (0.131, 0.849–0.882)
50–75% (n = 250, 1,036–4,082 px)	0.694 (0.190, 0.670–0.718)	0.562 (0.210, 0.535–0.588)	79.4 px (56.3, - 72.4–86.5)	0.876 (0.089, 0.865–0.887)
75–100% (n = 250, 4,082–13,651 px)	0.796 (0.144, 0.778–0.814)	0.682 (0.178, 0.660–0.705)	76.3 px (46.6, 70.5–82.1)	0.889 (0.084, 0.879–0.900)

**Table 2:** Ablation results evaluating the impact of key components of the proposed pipeline. Metrics include Dice, IoU, HD95, and pixel-wise AUC with associated variability measures. Removing post-processing or healthy reconstruction substantially reduces overlap accuracy and increases boundary error relative to the full model.

Configuration	Mean Dice (SD, 95% CI)	Mean IoU (SD, 95% CI)	Mean HD95 (SD, 95% CI)	Mean AUROC (SD, 95% CI)
Full model	0.596 (0.294, 0.578–0.614)	0.482 (0.280, 0.465–0.500)	80.5 px (62.7, 76.6–84.5)	0.861 (0.143, 0.852–0.870).
Without postprocessing	0.403 (0.257, 0.386–0.418)	0.287 (0.221, 0.273–0.300)	127 px (60.0, 124–131)	0.784 (0.136, 0.776–0.793)
Healthy prior only (inpainted with no encoder-decoder reconstruction)	0.224 (0.213, 0.211–0.237)	0.146 (0.168, 0.135–0.166)	126 px (60.6, 122–130)	0.495 (0.210, 0.482–0.508)

## Data Availability

The LiTS dataset used for evaluation is publicly available from its source (https://doi.org/10.1016/j.media.2022.102680) [8]. The training data consist of de-identified clinical CT scans governed by institutional data use and IRB policies. These were obtained from the University of Texas Southwestern Medical Center and contain protected health information. Due to institutional and regulatory restrictions, these data are not publicly available. All preprocessing scripts, model training code, inference pipelines, and evaluation routines used in this study will be made publicly available at the time of publication at: https://github.com/ejoshi1/liver_anomaly_detection.
